# Case of Hydatids of the Uterus Coincident with Pregnancy

**Published:** 1846-06

**Authors:** George Yeates Hunter

**Affiliations:** Surgeon, Margate


### RECORD OF MEDICAL SCIENCE.

*Case of Hydatids of the Uterus Coincident with Pregnancy.* By George Yeates Hunter, Esq., Surgeon, Margate.—On Monday, March 30th, about six o'clock, P. M., I was called to Mrs. B——, aged 23, in labour with her second child, Mr. W. H. Thornton, my assistant, who was engaged to attend, being absent.

Ou examination per vaginam, I found the os uteri dilated, and the head presenting; but the presentation was by no means distinct, and was ascertained with some difficulty, because a soft pulpy mass (evidently not the placenta) intervened between the presenting part and my finger.

After a few pains the membranes ruptured, at which time I heard a very peculiar crackling noise, and on again examining my patient, the infant's head had passed below the pulpy mass, which was now beyond my reach. The labour progressed very favourably, and the child, which was small, was born in less than two hours after my arrival. Mr. Thornton had arrived just before the child was born.

After waiting some time, it became necessary to remove the placenta. When the hand was introduced for that purpose, the uterus was found distended by what afterwards proved to be hydatids, measuring about three pints. They were in clusters, like bunches of grapes, some of them being the size of hot-house grapes, but others, and by far the greater number, of the size only of the common blue cluster grape.

When the placenta was detached, we found it lobulated, and of an average size; the hydatids appeared to have been attached to a part of its margin, as well as to the uterus. I have preserved a great portion of these hydatids and the placenta, of which I mean to make a preparation, for presentation to the College of Surgeons, should they think it worthy of a place in their museum.

The patient is going on very well; no untoward symptoms have yet shown themselves.

I am well aware that hydatids in the uterus have sometimes been met with, but I never saw, nor heard of, a case in which they were found in the pregnant uterus. I have shown the hydatids in this case to my medical friends in this neighbourhood, who consider the case unique and interesting.

It should be remarked, that there were in the above woman's case, no unusual symptoms during the progress of her pregnancy. I shall endeavour to keep this patient in view, for the purpose of ascertaining whether or not other hydatids are formed, either during, or in the absence of pregnancy.—*London Lancet.*

